# 16S-23S Internal Transcribed Spacer Region PCR and Sequencer-Based Capillary Gel Electrophoresis has Potential as an Alternative to High Performance Liquid Chromatography for Identification of Slowly Growing Nontuberculous Mycobacteria

**DOI:** 10.1371/journal.pone.0164138

**Published:** 2016-10-17

**Authors:** Shradha Subedi, Fanrong Kong, Peter Jelfs, Timothy J. Gray, Meng Xiao, Vitali Sintchenko, Sharon C-A Chen

**Affiliations:** 1 Centre for Infectious Diseases and Microbiology Laboratory Services, ICPMR – Pathology West, Westmead Hospital, Westmead, NSW, Australia; 2 Centre for Infectious Diseases and Microbiology – Public Health, Westmead Hospital, Westmead, New South Wales, Australia; 3 Department of Infectious Diseases and Microbiology, Concord Hospital, Concord, New South Wales, Australia; 4 Department of Clinical Laboratory, Peking Union Medical College Hospital, Chinese Academy of Medical Sciences, Beijing, China; 5 Centre for Infectious Diseases and Microbiology, Westmead Hospital and the Marie Bashir Institute for Infectious Diseases and Biosecurity, University of Sydney, Sydney, NSW, Australia; Universidad Autonoma Metropolitana, MEXICO

## Abstract

Accurate identification of slowly growing nontuberculous mycobacteria (SG-NTM) of clinical significance remains problematic. This study evaluated a novel method of SG-NTM identification by amplification of the mycobacterial 16S-23S rRNA internal transcribed spacer (ITS) region followed by resolution of amplified fragments by sequencer-based capillary gel electrophoresis (SCGE). Fourteen American Type Culture Collection (ATCC) strains and 103 clinical/environmental isolates (total n = 24 species) of SG-NTM were included. Identification was compared with that achieved by high performance liquid chromatography (HPLC), in-house PCR and 16S/ITS sequencing. Isolates of all species yielded a SCGE profile comprising a single fragment length (or peak) except for *M*. *scrofulaceum* (two peaks). SCGE peaks of ATCC strains were distinct except for peak overlap between *Mycobacterium kansasii* and *M*. *marinum*. Of clinical/environmental strains, unique peaks were seen for 7/17 (41%) species (*M*. *haemophilum*, *M*. *kubicae*, *M*. *lentiflavum*, *M*. *terrae*, *M*. *kansasii*, *M*. *asiaticum and M*. *triplex*); 3/17 (18%) species were identified by HPLC. There were five SCGE fragment length types (I–V) each of *M*. *avium*, *M*. *intracellulare* and *M*. *gordonae*. Overlap of fragment lengths was seen between *M*. *marinum* and *M*. *ulcerans*; for *M*. *gordonae* SCGE type III and *M*. *paragordonae; M*. *avium* SCGE types III and IV, and *M*. *intracellulare* SCGE type I; *M*. *chimaera*, *M*. *parascrofulaceum* and *M*. *intracellulare* SCGE types III and IV; *M*. *branderi* and *M*. *avium* type V; and *M*. *vulneris* and *M*. *intracellulare* type V. The ITS-SCGE method was able to provide the first line rapid and reproducible species identification/screening of SG-NTM and was more discriminatory than HPLC.

## Introduction

Slowly growing nontuberculous mycobacteria (SG-NTM) are environmental bacteria that can cause lung, lymph node, bone, skin and soft tissue, as well as disseminated, infections [1–4]. Infections are acquired following environmental exposure, and outbreaks of public health significance have been reported particularly in relation to contaminated water supplies [1, 5]. SG-NTM also colonize the airways of patients with chronic lung disease and are increasingly encountered in respiratory tract specimens [1, 6, 7]. The spectrum of recognized SG-NTM species (over 160) is ever widening (http://www.bacterio.net/mycobacterium.html) with evolving taxonomy, re-definition of species complex and identification of individual species within complexes [4, 8, 9]; an increasing number may cause human disease [1]. The most common pathogenic species or species groups include *Mycobacterium avium* complex (MAC; comprising *M*. *avium*, *M*. *intracellulare* and other species), *M*. *simiae* complex, *M*. *terrae* complex, *M*. *haemophilum*, *M*. *kansasii* and *M*. *marinum* [10]. Newer pathogenic species include *M*. *asiaticum*, *M*. *branderi*, *M*. *lentiflavum* and *M*. *triplex* [4]. Because of differences in clinical relevance and species-specific antimicrobial susceptibity and treatment regimens, accurate and rapid species identificaion of SG-NTM is of great importance [7, 9, 11].

**Fig 1:**
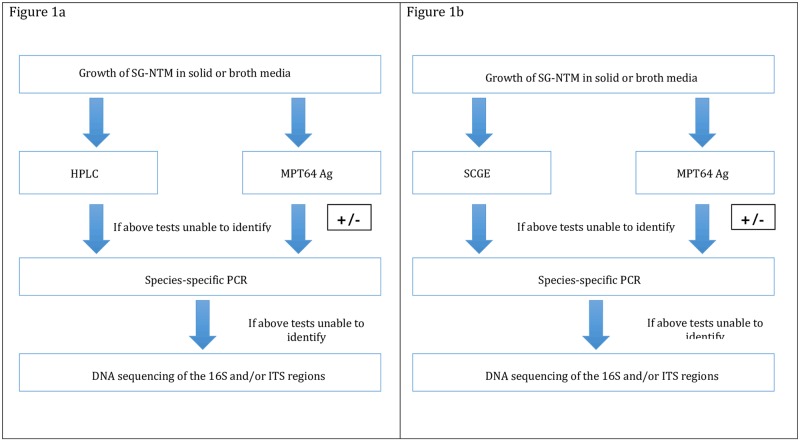
**a.** Algorithm used for identification of slowly growing nontuberculous mycobacteria in our laboratory. **b.** Proposed new algorithm incorporating SCGE for identification of slowly growing nontuberculous mycobacteria. **Species-specific PCR;** “TB/MAC” PCR, targeting *M. tuberculosis* [IS6110] and MAC [ITS]; “KGSA 1” PCR, targeting *M. gordonae* [ITS] and *M. kansasii* [16S]; “KGSA2” PCR, targeting *M. marinum/M.ulcerans* [ITS], *M. asiaticum* [ITS] and *M. szulgai* [ITS].

Traditionally, a combination of phenotypic, morphological and mycolic acid-based methods have been used to identify/screen SG-NTM. In our laboratory (Fig 1a), high performance liquid chromatography (HPLC) has been performed in parallel with the TB Ag MPT64 rapid test (SD Bioline, Standard Diagnostics, Suwon, Korea) for identification/screening of SG-NTM and exclusion of *Mycobacterium tuberculosis* [10]. However, this approach requires 1–2 days for identification and lacks discriminatory power to distinguish closely-related SG-NTM species [10]. Matrix-assisted laser desorption ionization—time of flight mass spectrometry although used routinely for bacterial identification currently is also unable to reliably distinguish closely related species [12–14]. Molecular methods such as probe-based assays, restriction enzyme analysis (REA) and gene sequencing are alternative approaches for identification of SG-NTM [10, 15–17]. In particular, sequencing of the 16S rRNA gene and the 16S-23S internal transcribed spacer (ITS) region has been often taken as the benchmark for species resolution [15, 16]. Multiplex PCR based assays targeting the 16S-23S rRNA to differentiate common NTM species have also been developed [18]. Sequencing of the heat shock protein 65 (*hsp65*) and beta subunit of RNA polymerase (*rpoB*) genes also yield good species discrimination [19, 20] yet no single target gene seems to be sufficiently discriminatory to accurately speciate all NTM.

Sequencer-based capillary gel electrophoresis (SCGE) is an alternative approach for fast, accurate organism identification/screening and has produced high resolution for species identification of *Clostridium difficile* [21, 22], *Vibrio* [23], *Nocardia* [24], rapidly growing mycobacteria (RGM) [25] and *M*. *tuberculosis* [26]. Unlike traditional gel electrophoresis, SCGE uses a 5’-end fluorescein-labelled primers and a DNA analyser, with resultant rapid and accurate resolution of amplicons. We recently applied SCGE to identify RGM and therein, had included several American Type Culture Collection (ATCC; Manassas, VA) SG-NTM strains (Table 1) to broadly illustrate the distinctiveness in profiles between these two groups of mycobacteria; however, no clinical SG-NTM isolates were studied [25]. Here we applied an ITS-SCGE assay to identify 103 clinical/environmental SG-NTM isolates and compared the results with those of a combination of HPLC, in-house multiplex PCR and 16S rRNA gene/ITS sequencing. The identification provided by DNA sequencing is definitive. We hypothesised that SCGE will improve the initial identification of SG-NTM and provide an alternative to HPLC. Fig 1b shows the proposed algorithm for identification of SG-NTM where SCGE is used as an alternative to HPLC.

**Table 1:** Sequencer capillary gel electrophoresis derived internal transcribed spacer-amplified fragment lengths (or peaks) of American Type Culture Collection reference strains of slowly growing nontuberculous mycobacteria.

*Reference strain ID*	*ATCC number*	*SCGE peak 1 (bp)*	*SCGE peak 2 (bp)*	*HPLC ID (Y/N)*
*M. gordonae*	14470	362^§^		N
*M. marinum*	927	364^§^		N
*M. kansasii*	12478	364^§^		Y
*M. shimodei*	27962	365^§^		N
*M. szulgai*	35799	366^§^		N
*M. avium*	25291	368^§§^		N
*M. asiaticum*	25276	369^§§^		N
*M. paraffinicum*	12670	370^§^		Y
*M. scrofulaceum*	19981	370^§^	372	Y
*M. intracellulare*	13950	371^§§^		N
*M. simiae*	25275	373^§^		Y
*M. xenopi*	19250	448^§^		Y
*M. nonchromogenicum*	19530	453^§^		Y
*M. terrae*	15755	456^§^		N

**Abbreviations**: ATCC, American Type Culture Collection; HPLC, high performance liquid chromatography; ID, identification; SCGE, sequencer capillary gel electrophoresis.

^§^ Previously reported by Gray et al [24]

^§§^ SCGE repeated in this study

## Materials and Methods

### Isolates

A total of 117 SG-NTM isolates representing 24 species/species complex referred to the New South Wales Mycobacterium Reference laboratory (Westmead Hospital, Sydney, Australia) from 1/1/2015-31/10/2016 were studied. These comprised of 101 clinical isolates, one environmental *M*. *gordona*e, and a “quality assurance” *M*. *kubicae*, isolates (Table 2). Fourteen ATCC strains (14 species) (Table 1) were also analysed and 3/14 ATCC strains previously analysed were repeated for comparison [25]. A clinical strain of *M*. *bovis* was included for comparison as a representative of *M*. *tuberculosis* complex.

**Table 2:** Sequencer based Capillary gel electrophoresis derived internal transcribed spacer-amplified fragment lengths (or peaks) of clinical, environmental and quality assurance SG-NTM species.

*Mycobacterium* species ID (no of isolates)	Average SCGE fragment length, bp	Range (bp)	SCGE peak unique (Y/N)	*Mycobacterium* species ID		
				HPLC ID (Y/N)	Multiplex / species-specific PCR ID (Y/N)	16S+/-ITS sequencing (Y/N)
*M. gordonae* SCGE type I (1)	360.86		Y	N	Y	N
*M. gordonae* SCGE type II (4)	361.14	361.11–361.35	Y	N	Y	N
*M. gordonae* SCGE type III (3)	361.60	361.46–361.70	N	N	Y	N
*M. paragordonae* (2)	361.65	361.65–361.66	N	N	N	Y
*M. gordonae* SCGE type IV (4)	362.11	361.90–362.22	Y	N	Y	N
*M. gordonae* SCGE type V (1)	363.29		Y	N	Y	N
*M. kansasii* (2)	363.89	363.85–363.92	Y	Y	Y	N
*M. ulcerans* (1)	364.06	364.06	N	N	N	N
*M. marinum* (3)	364.22	364.16–364.40	N	N	N	N
*M. kubicae* (1)	365.57	365.57	Y	N	N	Y
*M. avium* SCGE type I (2)	367.31	367.17–367.44	Y	N	Y	N
*M. avium* SCGE type II (1)	367.75		Y	N	Y	N
*M. avium* SCGE type III (13)	368.31	368.16–368.48	N	N	Y	N
*M. intracellulare* CGE type I (1)	368.44	368.44	N	N	Y	N
*M. avium* SCGE type IV (15)	368.58	368.49–368.60	N	N	Y	N
*M. avium* SCGE type V (2)	369.3	369.20–369.40	N	N	Y	N
*M. branderi* (1)	369.48	369.48	N	N	N	Y
*M. asiaticum* (1)	369.78	369.78	Y	N	Y	N
*M. chimaera* (3)	370.6	370.48–370.70	N	Y	N	Y
*M. parascrofulaceum* (2)	370.76	370.71–370.81	N	N	N	Y
*M. intracellulare* SCGE type II (12)	370.90	370.76–371.07	N	N	Y	N
*M.intracellulare* SCGE type III (16)	371.17	371.09–371.23	N	N	Y	N
*M. intracellulare* SCGE type IV (2)	371.60	371.52–371.66	Y	N	Y	N
*M. vulneris* (1)	371.88	371.88	N	N	N	Y
*M. intracellulare* SCGE type V (5)	372.07	371.94–372.20	N	N	Y	N
*M. lentiflavum* (1)	373.32	373.32	Y	Y	N	N
*M. triplex* (1)	374.51	374.51	Y	N	N	Y
*M. haemophilum* (1)	390.13	390.13	Y	N	N	Y
*M. terrae* (1)	459.11	459.11	Y	N	N	Y

**Abbreviations:** HPLC, High performance liquid chromatography; ID, identification; ITS, internal transcribed spacer; PCR, polymerase chain reaction; SG-NTM, Slow growing nontuberculous mycobacteria; SCGE, sequencer based capillary gel electrophoresis; r RNA, ribosomal ribonucleic acid; Y,Yes; N, No.

The clinical SG-NTM isolates (from 92 patients) were from sputum (n = 65 isolates), broncho-alveolar lavage fluid (n = 26), tissue (n = 8), and one isolate each from pleural fluid, and blood culture. Nine patients had two isolates cultured during the same illness episode, and one had three; these repeat isolates were analyzed to assess reproducibility of the SCGE procedure. Thirty-six isolates were *M*, *intracellulare*, 33 were *M*. *avium*, and 12, *M*. *gordonae*. Smaller numbers isolates of *M*. *marinum M*. *chimaera*, *M*. *kansasii*, *M*. *paragordonae*, *M*. *parascrofulaceum*, *M*. *asiaticum*, *M*. *branderi*, *M*. *haemophilum*, *M*. *lentiflavum*, *M*. *terrae*, *M*. *triplex*, *M*. *ulcerans and M*. *vulneris* were also examined (Table 2). Isolates had been received fresh in Mycobacterial growth Indicator Tube (MGIT), Middlebrook 7H10 or Lowenstein Jenson media. DNA extraction was performed on fresh cultures and extracts were stored in -20°C prior to analysis by SCGE.

### Identification of mycobacteria

SG-NTM were subjected to HPLC in conjunction with testing for TB Ag MPT64 (SD Bioline) [9, 27] (Fig 1a). For the purposes of the present study, all isolates were subjected to one or more in-house real—time multiplex PCR assays using primers and species-specific probes as appropriate, was then performed. Three different assays were used: 1) “TB/MAC PCR” with detects *M*. *tuberculosis* (IS*6110* target) or MAC (ITS-directed); 2) “KGSA-1 PCR” with targets for *M*. *kansasii* (16S), *M*. *gordonae* (ITS); and 3) “KGSA-2 PCR” with targets for *M*. *asiaticum* (ITS), *M*. *szulgai* (ITS), and *M*. *marinum*/*M*. *ulcerans* (ITS) [28]. For identification of *M*. *ulcerans*, a species-specific-PCR targeting the *M*. *ulcerans* insertion sequence IS*2404* was used to confirm identification [29]. A subset of isolates (n = 35) further underwent 16S rRNA gene and/or ITS sequencing as required (see below); all 35 could not be identified to species level by SCGE, HPLC or in-house PCR. Hence DNA sequencing was considered the reference method for identification. 16S rRNA gene and ITS sequences were compared with archived sequences of type strains in NCBI GenBank using the NCBI BLASTn (http://blast.ncbi.nlm.nih.gov/Blast.cgi?PROGRAM=blastn&PAGE_TYPE=BlastSearch&LINK_LOC=blasthome). All ATCC strains underwent 16S rRNA/ITS sequencing to verify their identity to exclude potential misclassification during storage.

### DNA extraction

DNA extraction was performed using the commercial InstaGene Matrix method (Bio-Rad Laboratories Inc, California, USA). Two to four colonies were retrieved from Middlebrook 7H11 agar and placed into 1 mL of sterile water, vortexed and then sonicated for 20 minutes. The resultant suspensions were centrifuged at 11,000 rpm for 1 minute. The supernatant was discarded and the residual pellet was resuspended in 200 mL of InstaGene Matrix (Bio-Rad), mixed by vortex and incubated at 56°C for 20 minutes. Samples were then boiled for 8 minutes and centrifuged (13,200 rpm) for 2 minutes prior to use. Samples were used within 24 hours or stored at -20°C. The resultant DNA extract was diluted to 1:100 using molecular-biology grade water (Eppendorf, North Ryde, Australia) prior to undergoing PCR.

### Amplification of the 16S-23S rRNA internal transcribed spacer region (ITS)

The *Mycobacteria* ITS region(s) were amplified prior to SCGE. The PCR Master mix comprised of 12.5 μL of hotstart master mix x2 (Qiagen, Doncaster, Vic.), 0.1 μL of 6-FAM labelled forward primer ITS1F (5’GTGCGGCTGGATCACCTCCT3’), 0.1 μL of ITS 1 reverse primer ITS1R (5’ AGCCTCCYACGTCCTTC[A/T]TCGGCT 3’), 5 μL of extracted DNA and molecular-biology grade water (Eppendorf) to make up a total volume of 25 μL. PCR conditions were 95°C for 15 minutes, 35 cycles of 95°C for 30 seconds, 62°C for 30 seconds, 72°C for 1 minute, followed by a further extension at 72°C for 10 minutes. Sequence analysis was performed by SCGE of the amplified ITS region. ITS sequencing was performed using the primer pair ITS2F and ITS2R: ITS2F forward primer 5’ GAAGTCGTAACAAGGTAGCCG 3’, ITS2R reverse primer 5’ GACAGCTCCCCGAGGC(A/T)TATCGCA 3’ (Sigma-Aldrich, Castle Hill, NSW). PCR conditions and mastermix (other than the primers) components were the same for PCR amplification prior to SCGE. A single band on agarose gel electrophoresis confirmed the presence of DNA amplicons.

### Sequencer-based capillary gel electrophoresis (SCGE)

Sequencer capillary gel electrophoresis and fragment analysis was performed as previously described [24, 25]. PCR fragment analysis was performed using the ABI 3730xl DNA analyzer employing a 48-capillary 50 cm POP-7 gel (Applied Biosystems, Forster City, USA). PCR products were diluted 1:30 with molecular biology-grade H_2_O (Eppendorf) to a final volume of 30 μL. Sample injection was at 1.6 kV over 15 seconds with a total running time of 6,200 seconds at 15 kV run voltage. A 20- to 1200-bp LIZ 1200 ladder (Chimerx, Madison, WI, USA) was the internal size control.

### SCGE interpretation and data analysis

Amplified fragments, represented by one or more peaks according to fragment size, were analyzed by Gene Mapper software (Applied Biosystems). A second software, Peak Scanner v1 (ThermoFisher Scientific, Cleveland, Ohio) was used to demonstrate consistency of peak position and size between different software. All peak with a height of <10% that of the highest peak in the individual profile were excluded as background signal rather than evidence of a major DNA fragment; when double peaks were observed ≤1.0 bp apart, only the larger peak was analyzed [24, 25]. Fragment lengths found in >10% of a species were called ‘common’ and fragment lengths found only in one species were defined as ‘unique’. Using IBM SPSS Statistics for Windows, Version 19.0, (Armonk, NY: IBM Corp) descriptive statistics was performed. The pattern of peaks was noted in addition to the number and numeric values of the fragments, and was rounded up or down to the nearest whole number for ATCC strains for “calling” a species. For clinical isolates, values were rounded to two decimal places; early experiments identified that this approach allowed for species identification with greater sensitivity without loss of resolution between known species (data not shown). Based on published work on the resolution of SCGE, peaks within +/- 0.10 bp difference from the average bp were classed as identical, whilst those that showed a difference of > 0.10 bp were considered dissimilar [30]; hence in our study if a peak between two known species was > 0.10 bp apart, the peaks were considered to be unique for their species.

Amongst clinical isolates, for certain species, small variations in bp lengths between strains of the same species were observed for individual SCGE peaks. Based on early experiments and optimizing resolution (data not shown), those fragments which had bp positions within +/- 0.3 from the average for that species were considered as having the same fragment length. If the length was outside +/- 0.3 bp, the fragments were classed as different fragment length types within that species.

### Ethics statement

This study describes a laboratory validation of a novel method for the specific identification of SG-NTM pathogens. Bacterial isolates were collected and stored as part of routine laboratory practice. No additional isolates were collected or stored for the purpose of this study. All bacterial samples were de-identified for the purpose of study experiments, data analysis and reporting. The study does not involve the collection or reporting of patient data and no patient intervention occurred with the results obtained.

## Results

Amplification of mycobacterial DNA with the primers ITS1F and ITS1R yielded a PCR product in all instances confirming good quality DNA for SCGE using our DNA extraction protocol.

### SCGE analysis of ATCC strains

**Fig 2:**
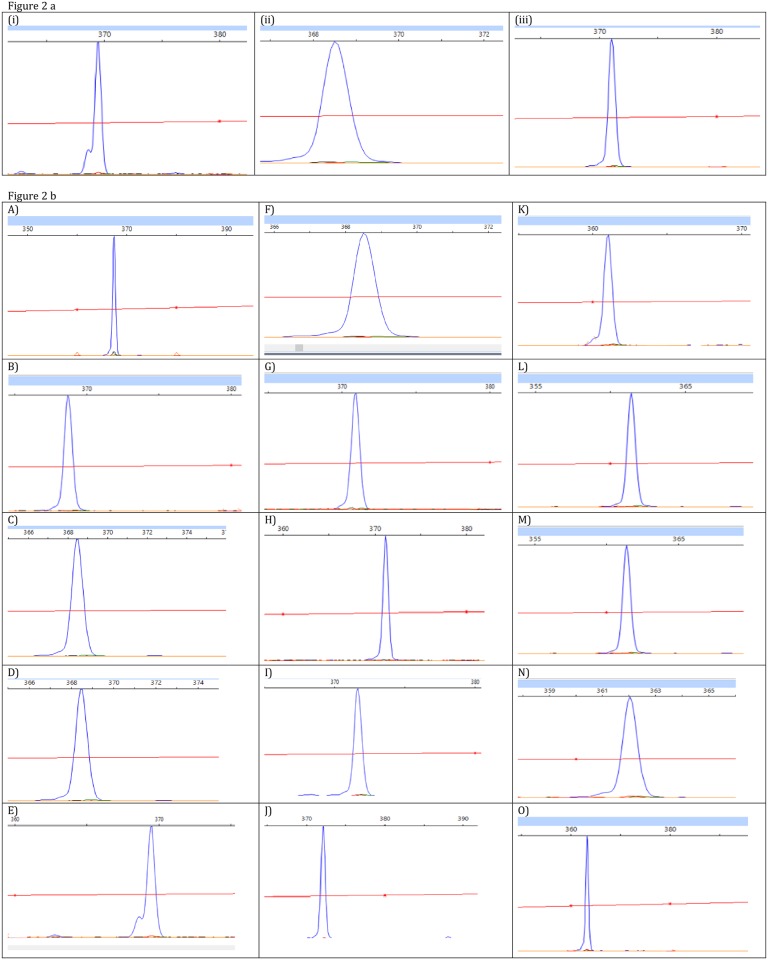
**a.** SCGE peaks of three representative ATCC strains. (i) *M*. *asiaticum* ATCC 25276 369 bp. (ii) *M*. *avium* ATCC 25291 368 bp. (iii) *M*. *intracellulare* ATCC 13950, 371 bp. **b**. Five SCGE fragment length types of *M*. *avium*, *M*. *gordonae* and *M*. *intracellulare*. All isolates were obtained from clinical specimen. A) *M*. *avium* SCGE type I 367.44 bp B) *M*. *avium* SCGE type II 367.75 bp C) *M*. *avium* SCGE type III 368.44 bp D) *M*. *avium* SCGE type IV 368.49 bp E) *M*. *avium* SCGE type V 369.2 bp F) *M*. *intracellulare* SCGE type I 368.44 bp G) *M*. *intracellulare* SCGE type II 370.96 bp H) *M*. *intracellulare* SCGE type III 371.17 bp I) *M*. *intracellulare* SCGE type IV 371.60 bp J) *M*. *intracellulare* SCGE type V 372.15 bp K) *M*. *gordonae* SCGE type I 360.86 bp L) *M*. *gordonae* SCGE type II 361.20 bp M) *M*. *gordonae* SCGE type III 361.50 bp N) *M*. *gordonae* SCGE type IV 362.0 bp O) *M*. *gordonae* SCGE type V 363.29 bp

Table 1 shows the sizes of the SCGE ITS fragment lengths (or peaks) for 14 ATCC strains in comparison with HPLC results and Fig 2a illustrates the SCGE profiles of three example strains. Fragment lengths ranged from 362 to 456 bp; 13 strains exhibited one peak and one (*M*. *scrofulaceum*), had two fragments (370 bp, 372 bp) [25]. Twelve strains generated distinct ITS-SCGE electropherograms, but peaks were rounded up to the nearest whole number (to set a strict criterion to distinguish between species). *M*. *marinum* ATCC 927 (364.18 bp) and *M*. *kansasii* ATCC 12478 (363.97 bp) displayed an indistinguishable peak at 364 bp (Table 1). In the present study, the profile of *M*. *avium* ATCC 25291 when analysed by the two different softwares (see “Methods”), and on two independent test occasions yielded a single peak at 368 bp. All ATCC stains were of their species designation by 16S rRNA gene/ITS sequencing (data not shown). HPLC identified six species including *M*. *kansasii* but not *M*. *marinum* (Table 1).

### Analysis of clinical and environmental isolates

S1 Fig. shows representative patterns of peaks of 17 species of clinical SG-NTM. Average SCGE ITS fragment sizes ranged from 361.65 to 459.11 bp. All isolates had profiles comprising single peaks. The fragment size for the *M*. *bovis* strain was 364.11 bp.

Of 101 clinical isolates, 21 (14 species) had electropherograms characterized by only one fragment type (see below) of single peak (average bp positions are shown in Table 2). These were *M*. *chimaera* (n = 3 isolates), *M*. *marinum* (n = 3), *M*. *kansasii* (n = 2), *M*. *paragordonae* (n = 2 isolates), *M*. *asiaticum* (n = 1), *M*. *branderi* (n = 1), *M*. *haemophilum* (n = 1), *M*. *kubicae* (n = 1), *M*. *lentiflavum* (n = 1), *M*. *parascrofulaceum* (n = 2), *M*. *terrae* (n = 1), *M*. *triplex* (n = 1), *M*. *ulcerans* (n = 1) and *M*. *vulneris* (n = 1). Unique species-specific peaks (separation of species by >/ = 0.10 bp difference [30]) was observed for seven species. The clearest separation (range 0.20- >50 bp from the next closest species-specific fragment) was seen for *M*. *haemophilum* (390.13 bp), *M*. *kubicae* (365.57 bp), *M*. *lentiflavum* (373.22 bp), *M*. *triplex* (374.51 bp) and *M*. *terrae* (459.11 bp) but distinct peaks were also evident for *M*. *asiaticum* and *M*. *kansasii*. HPLC identified 3/14 species (*M*. *kansasii*, *M*. *chimaera* and *M*. *lentiflavum*; Table 2).

Electropherograms however, were not readily distinguishable between the following species or species length types (see below): *M*. *chimera* and *M*. *parascrofulaceum*, *M*. *paragordonae*, and 3/12 *M*. *gordonae* strains, *M*. *branderi* and *M*. *vulneris* (Table 2). Multiplex PCR grouped isolates as *M*. *avium*, *M*. *intracellulare* and *M*. *gordonae* amongst others (Table 2), but was not able to speciate isolates not identified by either SCGE or HPLC. 16S/ITS sequencing was the only methodology able to identify *M*. *vulneris*, *M*. *chimaera*, *M*. *branderi* and *M*. *parascrofulaceum* (Table 2). Of note, the SCGE peak of *M*. *ulcerans* (364.06 bp) was indistinguishable from *M*. *bovis* (364.11 bp) using the criteria for species distinction in the study. The latter readily identifies as “*M*. *tuberculosis* complex” by HPLC.

For the three species—*M*. *avium*, *M*. *intracellulare* and *M*. *gordonae*—more than one SCGE fragment length type of single-peak electropherograms was observed with the lengths outside +/- 0.3 bp of average bp length for that species. There were five different SCGE length types (or “subtypes, assigned as length type I-V) for each of these species (Table 2); Fig 2b illustrates examples of these subtypes for *M*. *avium* and *M*. *intracellulare*. The SCGE pattern of four *M*. *gordonae* subtypes (length types I, II IV and V) were unique as were those of *M*. *avium* type I, *M*. *avium* type II and *M*. *intracellulare* type IV.

### Resolution of peak Overlaps

There were six instances of SCGE length overlap between species/species groups (Table 3). SCGE peaks of all *M*. *paragordonae* strains (average peak 361.65; range 361.65–361.66) overlapped with those of three *M*. *gordonae* fragment length type III strains (361.60; 361.46–361.70; Tables 2 and 3). Species identification for *M*. *gordonae* was achieved by the “KGSA 1” multiplex PCR whilst 16S sequencing identified *M*. *paragordonae*. Other species with fragment overlap were (i) *M*. *chimaera*, *M*. *parascrofulaceum* and *M*. *intracellulare* length types III and IV; (ii) *M*. *marinum* and *M*. *ulcerans*; (ii) *M*. *intracellulare* SCGE type I, and types III and IV of *M*. *avium* (iv) *M*. *avium* type V and *M*. *branderi*; and (v) *M*. *vulneris and M intracellulare* SCGE type V. Differentiation to species level for all the above was enabled by multiplex PCR or 16S/ITS sequencing (Table 3).

**Table 3:** Slowly growing mycobacterium species which demonstrated overlap of SCGE fragment lengths and the methods required to resolve species identification.

*Species 1*	*Species 2*	*Species 3*	*ID by PCR*	*ID by DNA sequencing*
*M. gordoane SCGE type III*	*M*. *paragordonae*	-	“KSGA 1” Multiplex PCR (*M*. *gordonae*)	16S rRNA and ITS Sequencing (*M*. *paragordonae*)
*M. avium SCGE type III*	*M*. *intracellulare* SCGE type I	*M*. *avium* SCGE type IV	“TB/ MAC” Multiplex PCR (for grouping as MAC)	ITS sequencing (for species)
*M. marinum*	*M*. *ulcerans*	-	*M*. *ulcerans* specific PCR	NA
*M. chimaera*	*M*. *parascrofulaceum*	*M*. *intracellulare* SCGE types III & IV	“TB/MAC” Multiplex PCR (for grouping as MAC)	16S rRNA/ITS sequencing (for species)
*M. avium SCGE type V*	*M*. *branderi*	-	“TB/MAC” Multiplex PCR (for grouping as MAC)	ITS sequencing (for species)
*M. vulneris*	*M*. *intracellulare* SCGE type V	-	“TB/MAC” Multiplex PCR (for grouping as MAC)	ITS sequencing (for species)

**Abbreviations:** ID, identification; ITS, internal transcribed spacer; ‘KGSA’, *Kansasii*/*gordonae*, MAC, *Mycobacterium avium* complex; NA, not applicable; PCR, polymerase chain reaction; SCGE, sequencer based capillary gel electrophoresis.

### Reproducibility and consistency

Reproducibility between operators and between experiments using the CGE technique has been demonstrated previously [24, 25]. In the present study we performed PCR and SCGE on two separate occasions for 10 (10%) isolates and obtained near identical results (data not shown). The mean difference in SCGE peaks obtained between the two software products, for clinical isolates was 0.05 (IQR 0.01–0.08). The mean difference in peaks observed amongst the nine duplicate and one triplicate isolates was 0.06 (IQR 0.05–0.08).

### Overall performance of SCGE in comparison with HPLC

**Fig 3:**
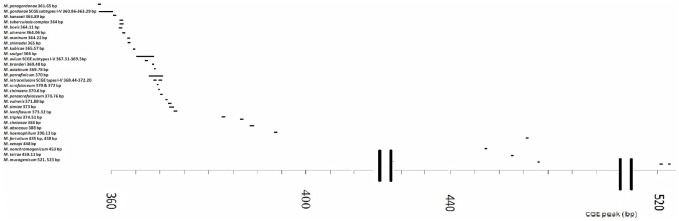
SCGE fragment lengths (or peaks) of 30 different *Mycoabacterium* species studied to date.

Overall, SCGE yielded unique fragments for 7/17 (41%) of clinical/environmental SG-NTM species studied (Table 2), and for an additional six species of ATCC strains (*M*. *nonchromogenicum*, *M*. *shimodei*, *M*. *scrofulaceum*, *M*. *szulgai* and *M*. *xenopi*), hence a total of 13/24 (54%) species studied. In comparison, HPLC identified 3/17 (18%) species of clinical isolates, a further five ATCC species (total 8/24; 33% species). SCGE could not identify *M*. *chimaera* and *M*. *paraffinicum*, which was achieved by HPLC. Of note, neither technique produced unique profiles for the more common groups of SG-NTM: *M*. *avium*, *M*. *intra*c*ellulare* and *M*. *gordonae*; although the SCGE peaks of 4/5 *M*. *gordonae*, 2/5 *M*. *avium* and 1/5 *M*. *intracellulare*, were unique (Table 2). Combining the results of the present study with those SCGE profiles of four species of RGM [25], the fragment lengths of various species are shown in Fig 3 to provide a more global representation of SCGE profiles of nontuberculous mycobacteria.

## Discussion

Accurate identification of SG-NTM in a diagnostic laboratory remains challenging due to absence of a robust, universal method for their speciation and in particular, to distinguish between closely related species [8]. Here, by studying a large number of SG-NTM isolates representing 24 species, we demonstrated that an ITS-targeted SCGE assay was able to rapidly and reliably identified 13 species and has potential as an alternative to HPLC-based identification of clinically relevant mycobacteria. That SCGE also has potential to distinguish between intra-species genetic types as demonstrated by different fragment length types within a number of SG-NTM species.

We previously observed that ITS SCGE patterns of ATCC strains of SG-NTM were distinct from those of other mycobacteria [25]. However, the utility of SCGE in the characterisation of clinical SG-NTM strains has not been explored. By re-analysing the 14 species of ATCC strains (identify confirmed by DNA sequencing), we confirmed the reproducibility of SCGE peaks over time. The single discrepant result with *M*. *avium* ATCC 25291 (a reproducible single peak in the present study vs. two peaks in the previous [25]) is likely to be caused by unsuspected contamination of the strain. We used strict criteria to reliably distinguish between species where fragment lengths were rounded to the nearest whole number, well above the limit of resolution of this technique (<0.06 bp) [30]. The two peaks for *M*. *scrofulaceum* ATCC 19981 are notable; further analysis of additional *M*. *scrofulaceum* strains would be worthwhile.

Of note, *M*. *marinum* ATCC 927 and *M*. *kansasii* ATCC 12478 produced a single 364 bp peak. However, when the peaks were rounded to two decimal places, they differed by 0.22 bp (364.18 and 363.96 bp, respectively; data not shown), being distinguishable using the criteria for clinical strains. *M*. *kansasii* is genetically closely related to *M*. *marinum* [31], and also to *M*. *tuberculosis* [25] and *M*. *bovis* (364.11 bp). Indeed, data indicate *M*. *kansasii* to represent the environmental ancestor of *M*. *tuberculosis* [31]. The inability of ITS-SCGE profiles on their own to readily differentiate between *M*. *kansaii* and *M*. *marinum* is broadly consistent with reports of ITS sequencing *per se* which is unable to resolve all NTM species [8, 32]. Hence any definitive identification method for SG-NTM should be evaluated using more than one genetic target to increase precision of identifications; one such target is the *rpoB* gene, which, by sequencing, may have superior discriminatory power over the ITS locus [19].

The complexity of SG-NTM identification is further borne out by the SCGE profiles for clinical/environmental isolates which illustrate the close evolutionary relationships of many SG-NTM. However, even using a less stringent rule to define uniqueness of peaks (bp rounded to two decimal places), certain closely related species had either very similar, or indistinguishable, fragment lengths, for example *M*. *kansasii*, *M*. *marinum* and *M*. *ulcerans*, where SCGE lengths were grouped at 364 bp. These species are phylogenetically closely related [4]. It has been suggested that *M*. *ulcerans* arose from *M*. *marinum* by horizontal gene transfer for a virulence plasmid [33]. In addition, *M*. *bovis*, included as a comparator species, was separated from *M*. *ulcerans* by only 0.05 bp. It is essential to distinguish SG-NTM from *M*. *tuberculosis* complex. At present, SCGE cannot differentiate *M*. *tuberculosis/M*. *bovis* from the above SG-NTM. Yet *M*. *tuberculosis* can be identified by HPLC in combination with the TB Ag MPT test (SD Bioline); this is a limitation of SCGE if it is to be offered as an alternative to HPLC-based identification. *M*. *bovis* (TB Ag MPT64 test negative) will require DNA sequencing for identification.

Fragment lengths of the *M*. *gordonae/M*. *paragordonae* group were generally indistinguishable; these two species share approximately 99% 16S rRNA gene sequence similarity [34]. Whilst they can be differentiated by growth characteristics (*M*. *paragordonae* grows at 25–30°C but not at 37°C, whereas *M*. *gordonae* grows at both temperatures), in our study and elsewhere, 16S rRNAgene /ITS sequencing were required for definitive identification [34]. Notably, SCGE profiles of *M*. paragordonae overlapped with those of *M*. *gordonae* length type III (see below).

Although some SCGE peaks of the more common species of MAC, *M*. *avium* and *M*. *intracellulare*, could be distinguished from one another (Table 2), profiles were not unique for either, nor was HPLC able to differentiate between *M*. *avium* and *M*. *intracellulare*. Of the other MAC species studied, *M*. *chimaera* (formerly *M*. *intracellulare* sequevar Mac-A [35]) and *M*. *vulneris*, HPLC correctly identified *M*. *chimaera* but its SCGE peak was not unique, overlapping with that of *M*. *parascrofulaceum* (Tables 2 and 3). *M*. *parascrofulaceum* belongs to the *M*. *simiae* complex [4] but for peak overlap with *M*. *chimaera* highlighted that the not closely related species could share a similar ITS fragment length. Neither HPLC, nor SCGE was able to identify *M*. *vulneris*, which required DNA sequencing [36].

Nonetheless, for clinical isolates it is noteworthy that SCGE clearly separated mycobacteria belonging to seven species. These species encompassed uncommon pathogens eg. *M*. *kubicae*, *M*. *scrofulaceum*, and *M*. *terrae*, as well as the more common *M*. *haemophilum*, *M*. *lentiflavum*, *M*. *asiaticum* and *M*. *triplex* [4]. HPLC was unable to identify *M*. *kubicae*, *M*. *triplex*, *M*. *haemophilum* and *M*. *terrae* for which SCGE had better discriminatory power. The ability to rapidly identify *M*. *haemophilum* is particularly relevant it is a significant pathogen in immunocompromised hosts [37]. That five species of *M*. *simiae* complex (*M*. *simiae*, *M*. *lentiflavum*, *M*. *kubicae*, *M*. *shimodei* and *M*. *triplex*) all had species-specific peaks further illustrate that SCGE can differentiate between closely related species.

A key finding of our study was that SCGE identified five different electropherogram patterns each for *M*. *avium*, *M*. *gordonae* and *M*. *intracellulare*. Within MAC, based on 16S rRNA gene analyses, there are four subspecies of *M*. *avium* and nine distinct species including *M*. *chimaera*, *M*. *intracellulare* and *M*. *vulneris* [38, 39]. It is unknown whether the five SCGE types of either *M*. *avium* or *M*. *intracellulare* identified here represent existing species or novel species. Subtypes of MAC are well studied by *hsp65* sequencing; comparison of SCGE with both *hsp65* and 16S rRNA gene sequencing, or of other targets such as *rpoB* would be of interest [38]. Genetic heterogeneity within *M*. *gordonae* species has also been described, particularly within the 5’ end of the 16S rRNA gene [40]. Based on PCR-REA of the *hsp65* gene, five subspecies of *M*. *gordonae* have been identified whilst PCR-REA of the *rpoB* gene, classified *M*. *gordonae* into four major clusters [41]. Again comparison of SCGE with PCR-REA gene analyses may help categorise SCGE types within *M*. *gordonae* against known subtypes. Whole genome sequencing has become more accessible and affordable to reference laboratories and may offer the ultimate level of resolution for identification and characterisation of Mycobacteria [9, 42].

The benefits of SCGE are that it is simple, reproducible, has minimum reagent requirements, requires only 6–8 hours to perform, and is not reliant on labour intensive agar gel techniques. Other advantage of SCGE is that results can be visualised across centers using accessible and standardised database. In comparison, HPLC is time consuming (total time 10–12 hours), requires considerable expertise and is best suited to a high throughput reference laboratory [27]. Although the running cost of HPLC is lower (AU$8.00 per isolate vs. AU$15.00 for SCGE), set up costs of HPLC are significant. In our laboratory, up front use of SCGE will reduce the need for species-specific PCR assays on many isolates, further reducing TAT and costs. Further expansion of the SCGE database using well characterised strains will gradually resolve the limitation of fragment overlap between closely related species and the ability to identify less common species. Importantly, the possibility that isolates from disparate geographic regions may occasionally exhibit differing electropherograms cannot be ruled out.

In conclusion, ITS-SCGE of the *Mycobacteria* can accurately identify clinically relevant SG-NTM and can distinguish between closely related species. It can potentially identify SCGE length types within species, particularly MAC, and lends itself as an appealing alternative to HPLC for the initial identification of SG-NTM. Collaborative efforts to develop a comprehensive SCGE-ITS database to complement existing identification approaches for SG-NTM are warranted.

## Supporting Information

S1 Fig Representative SCGE patterns of 17 species of clinical SG-NTM. A) *M*. *paragordonae* 361.65 bp B) *M*. *gordonae* SCGE type IV 362.03 bp C) *M*. *kansasii* 363.85 bp D) *M*. *ulcerans* 364.06 bp E) *M*. *marinum* 364.22 bp F) *M*. *kubicae* 365.57 bp G) *M*. *avium* SCGE type III 368.48 bp H) *M*. *branderi* 369.48 bp I) *M*. *asiaticum* 369.78 bp J) *M*. *chimaera* 370.60 bp K) *M*. *intracellulare* SCGE type V 371.98 bp L) *M*. *parascrofulaceum* 370.81 bp M) *M*. *vulneris* 371.88 bp N) *M*. *lentiflavum* 373.32 bp O) *M*. *triplex* 374.51 bp P) *M*. *haemophilum* 390.13 bp Q) *M*. *terrae* 459.11 bp
(TIF)
