# Supplementary figures and images for: 16S-23S Internal Transcribed Spacer Region PCR and Sequencer-Based Capillary Gel Electrophoresis has Potential as an Alternative to High Performance Liquid Chromatography for Identification of Slowly Growing Nontuberculous Mycobacteria
Source: PLoS One. 2016 Oct 17;11(10):e0164138. doi: 10.1371/journal.pone.0164138 (PMC5066948; doi:10.1371/journal.pone.0164138)

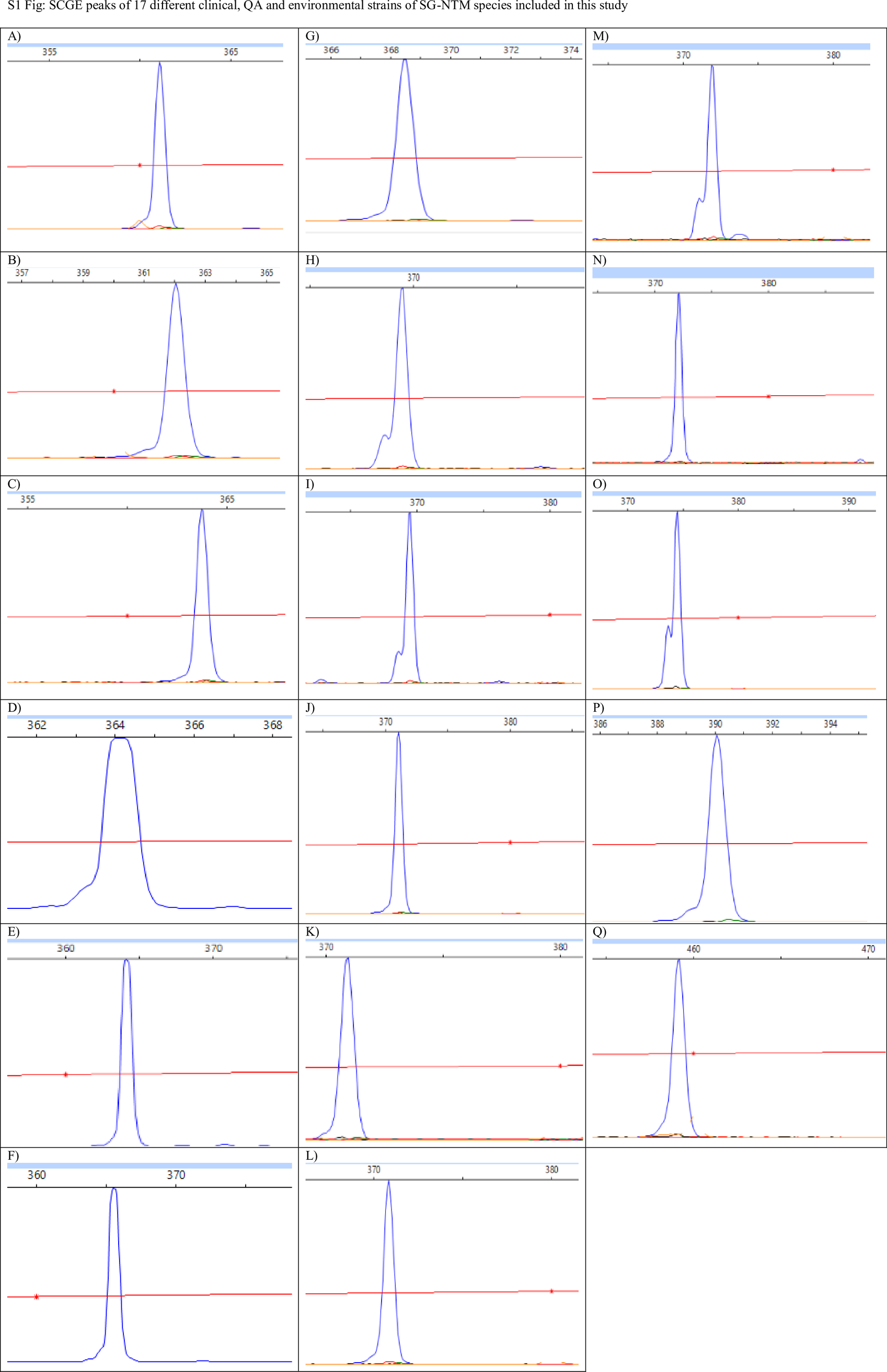

Supplement: S1 Fig — A) M. paragordonae 361.65 bp B) M. gordonae SCGE type IV 362.03 bp C) M. kansasii 363.85 bp D) M. ulcerans 364.06 bp E) M. marinum 364.22 bp F) M. kubicae 365.57 bp G) M. avium SCGE type III 368.48 bp H) M. branderi 369.48 bp I) M. asiaticum 369.78 bp J) M. chimaera 370.60 bp K) M. intracellulare SCGE type V 371.98 bp L) M. parascrofulaceum 370.81 bp M) M. vulneris 371.88 bp N) M. lentiflavum 373.32 bp O) M. triplex 374.51 bp P) M. haemophilum 390.13 bp Q) M. terrae 459.11 bp (TIF) [file pone.0164138.s001.tif]
